# Insecticide Resistance Dynamics and Spinosyn Cross-Resistance in *Megalurothrips usitatus* in Hainan, China

**DOI:** 10.3390/insects17060607

**Published:** 2026-06-09

**Authors:** Likui Wang, Linlin Yuan, Huihui Wu, Yutian Pu, Zhengke Peng, Fen Li, Pei Liang, Kun Zhang, Shaoying Wu

**Affiliations:** 1 Sanya Institute, China Agricultural University, Sanya 572024, China; 19937157228@163.com; 2 School of Breeding and Multiplication (Sanya Institute of Breeding and Multiplication), Hainan University, Sanya 572024, China; yllyll0105@126.com (L.Y.); 15720993676@163.com (H.W.); pyt_pu@163.com (Y.P.); lifen2010happy@sina.com (F.L.); 3 School of Tropical Agriculture and Forestry, Hainan University, Danzhou 571700, China; 4 School of Tropical Fruits and Vegetables, Hainan University, Baoting 572300, China; 5 Institute of Plant Protection, Guangdong Academy of Agricultural Sciences, Guangzhou 510640, China; zkpeng0827@163.com

**Keywords:** *Megalurothrips usitatus*, bioassay, insecticide resistance, spinosyns, cross-resistance

## Abstract

*Megalurothrips usitatus* is a major pest of cowpea in tropical regions, where frequent insecticide use has promoted the development of resistance. In this study, field populations of *M. usitatus* were collected from five major cowpea-producing areas in Hainan from 2023 to 2025, and their susceptibility to five commonly used insecticides was evaluated using a modified leaf-tube residual film method. Resistance levels varied among regions and insecticides, with southern populations generally showing higher LC_50_ values than central and northern populations. The tested neonicotinoid insecticide, acetamiprid, showed the highest resistance levels. By 2025, the resistance ratios of the Ledong, Sanya, and Lingshui populations to acetamiprid reached 298.48-, 139.60-, and 130.25-fold, respectively. Spinosyn resistance also increased rapidly, particularly in southern Hainan. The resistance ratio to spinetoram reached 110.78-fold in the Ledong population, while resistance to spinosad reached 37.06-fold in the Lingshui population. However, the absolute LC_50_ values of spinosad and spinetoram remained relatively low compared with those of acetamiprid and chlorfenapyr, indicating that these two spinosyn insecticides retained relatively high bioassay activity in laboratory bioassays. A significant positive correlation was detected between the LC_50_ values of spinosad and spinetoram across field populations (*r* = 0.8972, *p* < 0.0001), suggesting cross-resistance within the spinosyn class. These results indicate that resistance management for *M. usitatus* in Hainan should prioritize reducing the use of high-resistance-risk insecticides, avoiding consecutive applications of spinosyns, and designing region-specific rotation programs based on local resistance monitoring.

## Simple Summary

*Megalurothrips usitatus* is a major pest severely affecting cowpea production in tropical China. Intensive and frequent insecticide use has accelerated resistance development, threatening the sustainable control of this pest. In this study, we monitored the susceptibility of *M. usitatus* populations from five major cowpea-producing areas in Hainan from 2023 to 2025. The results showed that resistance was generally higher in southern Hainan than in central and northern regions. Acetamiprid, the tested neonicotinoid insecticide, showed the most severe resistance risk, with several southern populations reaching high-level resistance. Spinosyn resistance increased rapidly, and phenotypic evidence suggested cross-resistance between spinosad and spinetoram. These findings indicate that resistance management should focus on reducing the use frequency of acetamiprid and spinosyns, avoiding consecutive applications of insecticides with the same mode of action, and establishing region-specific rotation strategies for *M. usitatus* control in Hainan.

## 1. Introduction

Cowpea (*Vigna unguiculata* (L.) Walp.) is an important leguminous crop widely cultivated in tropical and subtropical regions because of its nutritional value and adaptability [1,2]. In many low-input farming systems, cowpea also contributes to household nutrition and food security [3]. The bean flower thrips, *Megalurothrips usitatus* (Bagnall), is one of the major insect pests of cowpea and can occur throughout the growing season [4]. Both adults and nymphs suck sap from the flowers, leaves, fruits and other parts of the host plants through their piercing-sucking mouthparts. During flowering, adults and nymphs often aggregate in flowers, where feeding can cause flower abscission, pod deformation, and yield loss [5,6]. As an insect vector, *M. usitatus* can also transmit viruses such as the peanut bud necrosis virus (PBNV) and tobacco streak virus (TSV) [7]. The secondary damage inflicted on plants during the virus transmission process is several times more severe than the direct feeding damage caused by the thrips, inducing a series of complex and abnormal physiological and biochemical responses in the host plants [8,9,10]. At present, chemical application remains the principal strategy for the field control of *M. usitatus*, frequently relying on diverse insecticide classes such as neonicotinoids (e.g., acetamiprid and thiamethoxam), pyrethroids (e.g., λ-cyhalothrin, permethrin, and bifenthrin), avermectins (e.g., abamectin and emamectin benzoate), pyrroles (e.g., chlorfenapyr), and spinosyns (e.g., spinosad and spinetoram) [11,12,13]. However, frequent insecticide applications in intensive cowpea production systems have increased selection pressure for resistance in *M. usitatus* field populations.

Repeated insecticide use is a major driver of resistance evolution in agricultural pests [14], and increasing resistance can substantially reduce the field performance of insecticides when the same compounds are used intensively [15].

This concern is directly relevant to *M. usitatus* in cowpea production, because thrips populations in vegetable and legume cropping systems are often exposed to repeated applications of a limited number of insecticide classes, including avermectins and spinosyns [11,12,13]. Similar susceptibility variation to multiple insecticides has also been reported in field populations of *Frankliniella occidentalis* in China [16]. High levels of resistance to spinosyn insecticides have been reported in several economically important thrips species. For example, *F. occidentalis* populations from Spanish greenhouses developed very high resistance to spinosad, and laboratory-selected strains showed extremely high resistance levels [17,18]. High-level spinosad resistance has also been reported in *Thrips tabaci* populations from Israel [19], while spinosad resistance in *Thrips palmi* has been associated with the G275E mutation in the nicotinic acetylcholine receptor α6 subunit and cytochrome P450-mediated detoxification [20]. In addition, rapid resistance development to spinetoram has been documented in *Thrips hawaiiensis* and field populations of *T. palmi*, further indicating that thrips can evolve resistance to spinosyns under sustained selection pressure [21,22]. These thrips-based examples provide a more relevant comparative context for resistance management in *M. usitatus* than examples from taxonomically and ecologically distant rice pests. In *M. usitatus*, monitoring data from 2016 to 2020 showed a rapid evolutionary trend in resistance to spinetoram in some populations [23].

Recent field surveys further revealed clear regional differentiation in insecticide susceptibility among *M. usitatus* populations in China. Peng et al. reported high resistance to spirotetramat in populations from Yunfu, Guangdong, whereas another study showed that some *M. usitatus* populations had lower susceptibility to spinetoram than to spinosad, with some populations reaching moderate resistance to spinetoram while remaining only low resistant to spinosad [12,23]. Large-scale maximum-dose bioassays and transcriptomic analyses have also confirmed reduced spinetoram efficacy and population-specific detoxification responses in *M. usitatus* [24,25]. Together, these findings indicate that resistance in *M. usitatus* is increasing and regionally heterogeneous, highlighting the need for continuous resistance monitoring to guide rational insecticide rotation and delay further resistance evolution [12,24,25].

Elucidating the current resistance status and evolutionary dynamics of target pests against commonly used insecticides is not only a prerequisite for developing scientific Insecticide Resistance Management (IRM) strategies, but also the phenotypic basis for deciphering the underlying molecular mechanisms of resistance. Although sporadic reports on the insecticide resistance of *M. usitatus* have previously emerged, there remains a lack of continuous and systematic spatiotemporal dynamic monitoring data specifically targeting the major producing areas in Hainan. Spinosad and spinetoram both belong to the spinosyn class [26], but the susceptibility relationship between these two compounds in field populations of *M. usitatus* remains insufficiently characterized. Therefore, this study investigated field populations of *M. usitatus* collected from five representative major cowpea-producing regions in Hainan Province (Haikou, Ledong, Sanya, Wuzhishan, and Lingshui) between 2023 and 2025. Using standardized laboratory bioassays, we systematically monitored variations in their susceptibility to five commonly applied insecticides: spinosad, spinetoram, emamectin benzoate, acetamiprid, and chlorfenapyr. The objectives were to characterize regional and temporal variation in resistance, compare LC_50_ and RR patterns among insecticides, and evaluate the susceptibility relationship between spinosad and spinetoram. The results provide information for region-specific resistance management of *M. usitatus* in Hainan.

## 2. Materials and Methods

### 2.1. Test Insects

The laboratory-susceptible strain of *M. usitatus* used in this study originated from a field population collected in 2017 at the agricultural research base of Hainan University. The population was continuously reared on fresh cowpeas in a standardized laboratory environment (26 °C, 65 ± 5% RH, and a 16 L:8 D photoperiod) without exposure to any chemical insecticides. To prevent pathogenic infection, cowpea pods used as feed were surface-disinfected with 0.5% sodium hypochlorite, followed by thorough rinsing with clean water and air-drying before use. Prior to the bioassays, vigorous female adults of uniform size and free of mechanical damage were selected from the rearing units using a self-made aspirator. These insects were subjected to a 2 h starvation period before testing to ensure consistency in feeding behavior or contact with the insecticide.

The field populations of *M. usitatus* used in this study were collected from five representative cowpea-producing regions in Hainan Province, China, during the primary growing seasons (November to April) from 2023 to 2025. The sampling sites covered the central, northern, and southern core cultivation areas of Hainan Island, specifically including Haikou (HK), Ledong (LD), Sanya (SY), Wuzhishan (WZS), and Lingshui (LS). Among these, the Lingshui population was added during 2024–2025 to broaden the coverage of southern Hainan, where cowpea production and insecticide use are intensive. Detailed information regarding the collection sites is provided in Table 1. To improve comparability among years, sampling was conducted within the same cowpea-producing locations or townships listed in Table 1. However, because cowpea cultivation, harvest schedules, and field availability varied among years, the exact individual fields sampled within each location were not necessarily identical each year. Therefore, the samples should be interpreted as annual field populations representing each cowpea-producing location rather than repeated collections from the same fixed field. Within each sampling area, the five-point sampling method was employed. Cowpea flowers, young leaves, and partial stems carrying *M. usitatus* nymphs and adults were collected and placed into 200-mesh breathable nylon bags. All samples were transported back to the laboratory within 24 h for bioassays. The toxicity bioassays were conducted directly using F_0_ field-collected adults, and the field populations were not reared for additional generations before testing. Before bioassays, adult thrips were identified under a stereomicroscope according to morphological characteristics, and only confirmed *M. usitatus* female adults were selected for testing.

### 2.2. Test Insecticides

The following five commercial insecticide formulations were used for the bioassays in this study: 5% emamectin benzoate microemulsion (ME) (Huizhou Yinlong Technology Co., Ltd., Huizhou, China), 10% acetamiprid emulsifiable concentrate (EC) (Shandong Zhongxinkenong Biotechnology Co., Ltd., Jinan, China), 10% spinosad suspension concentrate (SC) (Anhui Huaxing Chemical Industry Co., Ltd., Ma’anshan, China), 60 g/L spinetoram suspension concentrate (SC) (Corteva Agriscience China Co., Ltd., Shanghai, China), and 240 g/L chlorfenapyr suspension concentrate (SC) (BASF China Co., Ltd., Shanghai, China). All insecticides were utilized within their shelf-life periods, and dilutions were prepared with distilled water containing 0.1% Tween-80 as needed for the experiments.

### 2.3. Bioassay Method

The toxicity of the five insecticides against *M. usitatus* laboratory and field populations was evaluated using a modified leaf-tube residual film method [27]. Insecticides were diluted into five concentrations using distilled water containing 0.1% (*v*/*v*) Tween-80. Distilled water containing 0.1% Tween-80 served as the control. Preliminary bioassays were conducted to determine concentration ranges that produced approximately 10–90% mortality for each insecticide and population. Centrifuge tubes (1.5 mL) with ventilated caps (5 mm holes) were immersed in the respective solutions for 1 h and air-dried in a fume hood. Concurrently, surface-sterilized fresh cowpea pods were cut into 5 mm segments, dipped in the corresponding insecticide solutions for 30 s, and air-dried at room temperature. Distilled water containing 0.1% Tween-80 served as the control. For the bioassay, approximately 20 female adults were aspirated into each treated tube along with a treated cowpea segment. The tubes were sealed with gauze and capped. Each concentration, including the control, was replicated three times. Mortality was assessed at 48 h post-treatment; insects exhibiting no movement when gently probed with a fine brush were scored as dead. Bioassays were repeated when control mortality exceeded 10%. When control mortality ranged from 5% to 10%, mortality was corrected using Abbott’s formula.

### 2.4. Data Analysis

Probit regression analysis was conducted using POLO-Plus 2.0 software. The software was used to calculate the median lethal concentration (LC_50_), 95% fiducial limits (95% FL), slope of the toxicity regression line with its standard error (Slope ± SE), and Chi-square (χ^2^) values for each tested insecticide against the different geographical populations of *M. usitatus*. To quantify the resistance level, the resistance ratio (RR) was calculated using the LC_50_ value of the laboratory-susceptible strain as the baseline, defined as RR = LC_50_ of the field population/LC_50_ of the susceptible strain. According to the 2024 National Monitoring, Assessment and Management Strategies for Agricultural Pest Resistance (China) [28], the resistance levels of *M. usitatus* to insecticides were classified into four categories: susceptibility (0 < RR ≤ 5), low-level resistance (5 < RR ≤ 10), moderate-level resistance (10 < RR ≤ 100), and high-level resistance (RR > 100). This classification has also been used in national monitoring reports involving *F. occidentalis* in China. To assess the potential cross-resistance between different insecticides, the LC_50_ values of all tested populations were log-transformed. Pearson correlation analysis was performed using IBM SPSS Statistics 24. A significant positive correlation was considered phenotypic evidence suggesting potential cross-resistance between the paired insecticides.

## 3. Results

### 3.1. Resistance Dynamics of M. usitatus Field Populations to Different Insecticides

#### 3.1.1. Emamectin Benzoate

During the continuous monitoring period from 2023 to 2025, this study systematically evaluated the variation trends in susceptibility and the spatial distribution characteristics of *M. usitatus* field populations to emamectin benzoate (Table 2). The LC_50_ of the laboratory-susceptible strain to emamectin benzoate was 4.21 mg·L^−1^, which was used as the baseline for calculating resistance ratios. During 2023–2025, the LC_50_ values of field populations ranged from 5.11 to 128.19 mg·L^−1^, and the corresponding RR values ranged from 1.21- to 30.45-fold. According to the resistance classification criteria, the monitored populations were classified as susceptible, low-level resistant, or moderately resistant to emamectin benzoate. Resistance to emamectin benzoate was generally higher in southern Hainan than in the central and northern populations. In Ledong, the LC_50_ increased from 63.92 mg·L^−1^ in 2023 to 103.33 mg·L^−1^ in 2024 and 128.19 mg·L^−1^ in 2025, with the RR increasing from 15.18- to 30.45-fold. The Sanya population also showed an increase in resistance, with the RR rising from 7.77-fold in 2023 to 21.17-fold in 2024 and 27.45-fold in 2025. In Lingshui, the LC_50_ increased from 55.35 mg·L^−1^ in 2024 to 106.54 mg·L^−1^ in 2025, and the RR increased from 13.15- to 25.31-fold. These three southern populations were all classified as moderately resistant by 2025. In contrast, the Haikou and Wuzhishan populations showed lower resistance levels. In Haikou, the LC_50_ increased from 5.11 mg·L^−1^ in 2023 to 26.21 mg·L^−1^ in 2025, with the RR increasing from 1.21- to 6.23-fold. In Wuzhishan, the LC_50_ increased from 12.16 to 32.33 mg·L^−1^ during the same period, and the RR increased from 2.89- to 7.68-fold. Both populations remained susceptible or showed low-level resistance during most of the monitoring period. Overall, resistance to emamectin benzoate increased during 2023–2025, but the magnitude of increase differed among regions. Southern populations showed moderate resistance, whereas Haikou and Wuzhishan remained at lower resistance levels.

#### 3.1.2. Acetamiprid

The LC_50_ of the laboratory-susceptible strain to acetamiprid was 6.16 mg·L^−1^, which was used as the baseline for calculating resistance ratios (Table 3). During 2023–2025, the LC_50_ values of field populations ranged from 66.49 to 1838.63 mg·L^−1^, and the corresponding RR values ranged from 10.79- to 298.48-fold, indicating that all monitored populations had developed moderate or high-level resistance to acetamiprid. Compared with the other tested insecticides, acetamiprid showed the highest overall resistance level, with particularly high LC_50_ values in southern Hainan. In the Ledong population, the LC_50_ increased from 353.65 mg·L^−1^ in 2023 to 535.10 mg·L^−1^ in 2024 and further to 1838.63 mg·L^−1^ in 2025, while the RR increased from 57.41- to 298.48-fold, reaching the high-resistance level in 2025. The Sanya population showed a similar increase, with the LC_50_ rising from 306.26 to 859.93 mg·L^−1^ and the RR increasing from 49.72- to 139.60-fold during the same period. In Lingshui, the LC_50_ increased from 530.55 mg·L^−1^ in 2024 to 802.36 mg·L^−1^ in 2025, with the RR increasing from 86.13- to 130.25-fold. Thus, Ledong, Sanya, and Lingshui all reached high-level resistance to acetamiprid by 2025. In contrast, the Haikou and Wuzhishan populations remained within the moderate-resistance range, although their LC_50_ values were still clearly higher than those of the susceptible strain. The RR of Haikou increased from 10.79-fold in 2023 to 46.10-fold in 2025, while that of Wuzhishan ranged from 31.97- to 41.49-fold. Overall, acetamiprid resistance was detected across all monitored regions, and the highest resistance levels occurred in the southern populations.

#### 3.1.3. Chlorfenapyr

The LC_50_ of the laboratory-susceptible strain to chlorfenapyr was 11.43 mg·L^−1^, which served as the baseline for resistance-ratio calculation (Table 4). During the monitoring period, the LC_50_ values of field populations ranged from 86.96 to 956.11 mg·L^−1^, and the RR values ranged from 7.61- to 83.65-fold, showing low to moderate resistance to chlorfenapyr among Hainan populations of *M. usitatus*. Resistance was generally higher in the southern populations than in the central and northern populations. The Ledong population showed the highest chlorfenapyr tolerance, with the LC_50_ increasing from 225.73 mg·L^−1^ in 2023 to 531.68 mg·L^−1^ in 2024 and 956.11 mg·L^−1^ in 2025, corresponding to an increase in RR from 19.75- to 83.65-fold. The Sanya population also maintained relatively high resistance levels, with the LC_50_ increasing from 285.77 to 736.30 mg·L^−1^ and the RR increasing from 25.00- to 64.42-fold from 2023 to 2025. In Lingshui, the LC_50_ increased from 123.90 mg·L^−1^ in 2024 to 349.60 mg·L^−1^ in 2025, and the RR increased from 10.84- to 30.59-fold. By comparison, the Haikou and Wuzhishan populations showed lower resistance levels or slower increases. The RR of Haikou increased from 18.21- to 28.15-fold, while that of Wuzhishan increased from 7.61-fold in 2023 to 24.99-fold in 2025. Overall, chlorfenapyr resistance increased during 2023–2025, especially in Ledong and Sanya, but no population reached the high-resistance level.

#### 3.1.4. Spinosad

The resistance monitoring results from 2023 to 2025 (Table 5) show that, unlike the widespread high-level resistance observed for acetamiprid, the resistance evolution of *M. usitatus* to the spinosyn insecticide spinosad is currently in a stage characterized by concurrent rapid accumulation and regional differentiation. The LC_50_ of the long-term, closed-system laboratory-reared susceptible strain to spinosad was 0.35 mg·L^−1^ (0.22–0.53), indicating that this insecticide exhibits high absolute toxicity at the susceptibility baseline. Across the monitored field populations, the LC_50_ values ranged from 0.79 to 12.97 mg·L^−1^, and the corresponding RR values ranged from 2.26- to 37.06-fold. These results showed that resistance to spinosad increased during 2023–2025, particularly in southern Hainan, but the absolute LC_50_ values remained relatively low compared with those of acetamiprid and chlorfenapyr. In the Ledong population, the LC_50_ increased from 2.41 mg·L^−1^ in 2023 to 4.53 mg·L^−1^ in 2024 and 11.31 mg·L^−1^ in 2025, with the RR increasing from 6.89- to 32.31-fold. The Sanya population showed a similar trend, with the LC_50_ increasing from 1.84 to 9.05 mg·L^−1^ and the RR increasing from 5.26- to 25.86-fold from 2023 to 2025. In Lingshui, the LC_50_ increased from 4.23 mg·L^−1^ in 2024 to 12.97 mg·L^−1^ in 2025, corresponding to an increase in RR from 12.09- to 37.06-fold, which was the highest RR recorded for spinosad in this study. In contrast, the Haikou and Wuzhishan populations showed lower LC_50_ values and lower resistance levels. By 2025, their LC_50_ values were 2.04 and 2.50 mg·L^−1^, respectively, with RR values of 5.83- and 7.14-fold. Overall, spinosad resistance increased most clearly in the southern populations, whereas Haikou and Wuzhishan remained at relatively low resistance levels; however, the low absolute LC_50_ values suggest that spinosad still retained relatively high bioassay toxicity against *M. usitatus*.

#### 3.1.5. Spinetoram

During the continuous monitoring period from 2023 to 2025, this study systematically evaluated the spatiotemporal evolution characteristics of susceptibility in *M. usitatus* field populations to spinetoram, a second-generation spinosyn insecticide (Table 6). The LC_50_ of the laboratory-susceptible strain to spinetoram was 0.09 mg·L^−1^, which was the lowest baseline LC_50_ among the five tested insecticides (Table 6). In field populations, the LC_50_ values ranged from 0.79 to 9.97 mg·L^−1^, whereas the RR values ranged from 8.78- to 110.78-fold, indicating that spinetoram resistance should be interpreted by considering both RR values and absolute LC_50_ values. Resistance increased most clearly in southern Hainan. In the Ledong population, the LC_50_ increased from 1.82 mg·L^−1^ in 2023 to 4.16 mg·L^−1^ in 2024 and 9.97 mg·L^−1^ in 2025, while the RR increased from 20.22- to 110.78-fold, reaching the high-resistance level in 2025. The Lingshui population also showed a marked increase, with the LC_50_ rising from 2.22 mg·L^−1^ in 2024 to 7.86 mg·L^−1^ in 2025 and the RR increasing from 24.67- to 87.33-fold. In Sanya, the LC_50_ increased from 1.33 to 4.08 mg·L^−1^ from 2023 to 2025, and the RR increased from 14.78- to 45.33-fold. The Haikou and Wuzhishan populations showed lower LC_50_ values than the southern populations. In Haikou, the LC_50_ increased from 0.79 mg·L^−1^ in 2024 to 1.39 mg·L^−1^ in 2025, with the RR increasing from 8.78- to 15.44-fold. In Wuzhishan, the LC_50_ increased from 0.82 mg·L^−1^ in 2023 to 2.94 mg·L^−1^ in 2024 and then remained at 2.79 mg·L^−1^ in 2025, with RR values ranging from 9.11- to 32.67-fold. Overall, spinetoram resistance increased rapidly, especially in Ledong and Lingshui, but its absolute LC_50_ values remained relatively low compared with those of acetamiprid and chlorfenapyr, suggesting that spinetoram still showed high bioassay activity despite the increase in RR.

### 3.2. Annual and Regional Variation in LC_50_ Values and Resistance Levels Among Insecticides

**Figure 1:**
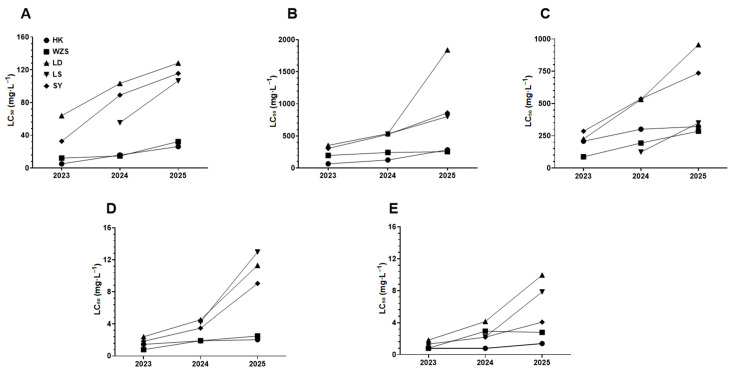
Temporal changes in LC_50_ values of *M. usitatus* field populations to five insecticides from 2023 to 2025. (**A**) Emamectin benzoate; (**B**) acetamiprid; (**C**) chlorfenapyr; (**D**) spinosad; (**E**) spinetoram. Each data point represents the LC_50_ value of a field population in a given year. HK, Haikou; WZS, Wuzhishan; LD, Ledong; LS, Lingshui; SY, Sanya. Note that y-axis scales differ among panels.

Annual monitoring from 2023 to 2025 showed clear year-to-year and regional differences in the LC_50_ values of *M. usitatus* field populations to the five tested insecticides (Figure 1, Table 2, Table 3, Table 4, Table 5 and Table 6). Overall, populations from southern Hainan, including Ledong, Sanya, and Lingshui, generally showed higher LC_50_ values than those from Haikou and Wuzhishan, although the magnitude of change differed among insecticides. For emamectin benzoate, LC_50_ values increased mainly in the southern populations, with Ledong, Sanya, and Lingshui reaching moderate resistance by 2025. Acetamiprid showed the most pronounced increase in both LC_50_ and RR values. In 2025, the LC_50_ values of acetamiprid reached 1838.63 mg·L^−1^ in Ledong, 859.93 mg·L^−1^ in Sanya, and 802.36 mg·L^−1^ in Lingshui, corresponding to high-level resistance in these southern populations. Chlorfenapyr also showed increasing LC_50_ values, especially in Ledong and Sanya, where the LC_50_ values reached 956.11 and 736.30 mg·L^−1^ in 2025, respectively; however, the RR values remained within the moderate-resistance range. In contrast, the LC_50_ values of spinosad and spinetoram remained much lower than those of acetamiprid and chlorfenapyr, despite clear increases during the monitoring period. The highest LC_50_ values recorded for spinosad and spinetoram were 12.97 and 9.97 mg·L^−1^, respectively, while their RR values reached 37.06-fold and 110.78-fold. These results indicate that acetamiprid showed the greatest reduction in susceptibility, chlorfenapyr and emamectin benzoate showed regional resistance accumulation, and spinosyn insecticides showed higher RR values in later sampling years while maintaining relatively low LC_50_ values in laboratory bioassays.

### 3.3. Relationship Between Spinosad and Spinetoram Susceptibility

**Figure 2:**
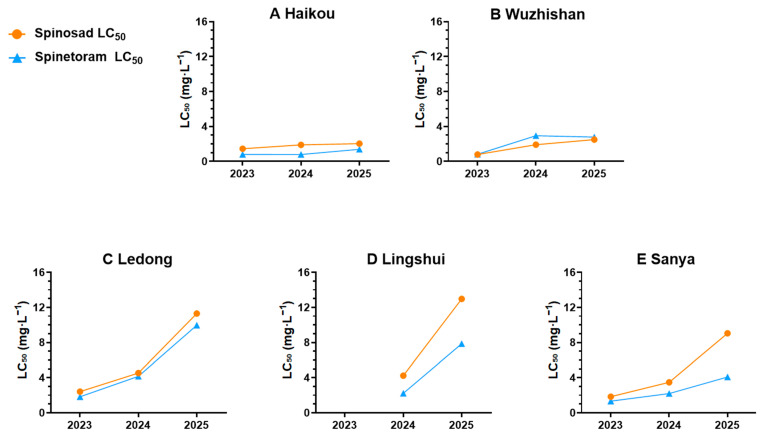
Temporal changes in LC_50_ values of *M. usitatus* field populations to spinosad and spinetoram from 2023 to 2025. (**A**) Haikou; (**B**) Wuzhishan; (**C**) Ledong; (**D**) Lingshui; (**E**) Sanya. Each point represents the LC_50_ estimate of one field population in a given year. The Lingshui population was monitored from 2024 to 2025.

**Figure 3:**
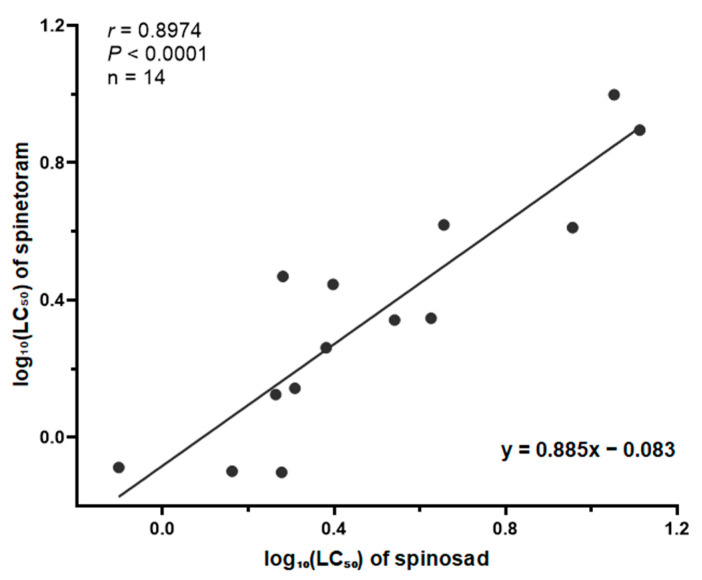
Correlation between log_10_-transformed LC_50_ values of spinosad and spinetoram in field populations of *M. usitatus*. Each point represents one population-year combination from 2023 to 2025. LC_50_ values were expressed in mg·L^−1^ before log_10_ transformation. The solid line represents the fitted linear regression. Pearson correlation analysis showed a significant positive correlation between the two insecticides (*r* = 0.8972, *p* < 0.0001, *n* = 14).

Because spinosad and spinetoram are both spinosyn insecticides [26], the relationship between susceptibility to these two compounds was further examined using the monitoring data from 2023 to 2025 (Table 5 and Table 6; Figure 2 and Figure 3). The two insecticides showed similar spatial and temporal patterns. Populations from southern Hainan, especially Ledong, Lingshui, and Sanya, generally showed higher LC_50_ and RR values than those from Haikou and Wuzhishan, and resistance to both insecticides increased during the monitoring period. In the Ledong population, the RR to spinosad increased from 6.89-fold in 2023 to 32.31-fold in 2025, while the RR to spinetoram increased from 20.22-fold to 110.78-fold during the same period. In Lingshui, the RR increased from 12.09- to 37.06-fold for spinosad and from 24.67- to 87.33-fold for spinetoram from 2024 to 2025. Pearson correlation analysis based on log10-transformed LC_50_ values showed a significant positive correlation between spinosad and spinetoram susceptibility responses across all monitored populations (*r* = 0.8972, *p* < 0.0001), indicating that populations with reduced susceptibility to spinosad also tended to show reduced susceptibility to spinetoram. Although the RR values of spinetoram were generally higher than those of spinosad, this difference was partly associated with the lower LC_50_ baseline of the susceptible strain to spinetoram. The LC_50_ of the susceptible strain was 0.09 mg·L^−1^ for spinetoram and 0.35 mg·L^−1^ for spinosad. For example, in the Ledong population in 2025, the absolute LC_50_ values of spinosad and spinetoram were 11.31 and 9.97 mg·L^−1^, respectively. These results suggest a positive association between spinosad and spinetoram susceptibility in field populations of *M. usitatus*, while also showing that both insecticides retained relatively low LC_50_ values compared with acetamiprid and chlorfenapyr.

## 4. Discussion

The three-year monitoring data showed marked geographical variation in the susceptibility of *M. usitatus* field populations in Hainan. Southern populations, particularly those from Ledong, Sanya, and Lingshui, generally had higher LC_50_ and RR values than the Haikou and Wuzhishan populations, although the extent of this difference varied among insecticides. This pattern was most evident for acetamiprid, but regional increases were also observed for emamectin benzoate, chlorfenapyr, spinosad, and spinetoram. Previous studies have shown that *M. usitatus* is closely associated with cowpea production in Hainan and southern China, and that its population dynamics are affected by host availability, temperature, and local production systems [4,6,10,11]. Earlier field evaluations and resistance studies also indicated that susceptibility to commonly used insecticides may differ among geographical populations of *M. usitatus* [12,23,24,25]. The Guangdong populations reported by Peng et al. and the Hainan populations examined here differed in geographic origin, tested insecticides, cropping systems, and likely insecticide-use histories [12]. Therefore, the Guangdong results provide useful comparative evidence but should not be directly generalized to Hainan. The higher resistance levels observed in southern Hainan may therefore reflect the combined effects of intensive winter–spring cowpea production, frequent insecticide applications, and favorable conditions for thrips development. The present bioassays, however, cannot separate the relative contributions of climate, cropping intensity, pesticide-use history, and population movement. These results are better interpreted as evidence of regional resistance differentiation under local production conditions rather than as the effect of a single ecological factor.

Acetamiprid showed the greatest reduction in susceptibility among the five tested insecticides. By 2025, the Ledong, Sanya, and Lingshui populations had reached high-level resistance, and the Ledong population had the highest LC_50_ and RR values recorded in this study. Because acetamiprid was the only neonicotinoid tested here, the result should be described as high resistance to the tested neonicotinoid compound rather than generalized to all neonicotinoids. Even so, the high LC_50_ values in southern populations suggest that repeated exposure to acetamiprid has imposed strong selection pressure in these production areas. More directly relevant evidence is available from thrips and legume-associated systems. *M. usitatus* populations collected from lablab bean have been evaluated against acetamiprid and other commonly used insecticides, and recent functional work involving thrips nicotinic acetylcholine receptor subunits showed that the V65I mutation can reduce sensitivity to neonicotinoids and sulfoxaflor [13,29]. The present study did not examine target-site mutations or detoxification genes, but the high resistance level detected for acetamiprid indicates that mechanism-based monitoring will be needed in the southern populations.

The results for spinosad and spinetoram require a more balanced interpretation. Resistance ratios increased during 2023–2025, especially in southern Hainan. Spinetoram resistance reached 110.78-fold in Ledong, and spinosad resistance reached 37.06-fold in Lingshui. These findings are consistent with previous reports showing that *M. usitatus* can develop reduced susceptibility to spinetoram under field selection [23,25]. At the same time, RR values alone may overstate the practical loss of toxicity when the susceptible baseline is very low. In this study, the LC_50_ values of the susceptible strain were 0.35 mg·L^−1^ for spinosad and 0.09 mg·L^−1^ for spinetoram. The much lower baseline for spinetoram partly explains why its RR values were higher than those of spinosad. For example, in the Ledong population in 2025, the RR values differed markedly between spinosad and spinetoram, but their absolute LC_50_ values were close, at 11.31 and 9.97 mg·L^−1^, respectively. This suggests that spinosyn resistance is increasing, but these two insecticides still showed relatively high bioassay toxicity compared with acetamiprid and chlorfenapyr. Recent analyses of spinosyn resistance also emphasize that field resistance should be interpreted in relation to both resistance ratios and field-relevant toxicity, because many field-selected cases show variable resistance levels and do not necessarily correspond to complete loss of control [30].

The significant positive correlation between log_10_-transformed LC_50_ values of spinosad and spinetoram suggests a possible association in susceptibility responses within the spinosyn class. However, this result should be interpreted cautiously because the analysis was based on pooled population-year data. Region, sampling year, and shared spinosyn selection pressure may all have contributed to the observed correlation. Therefore, the correlation provides phenotypic evidence suggesting possible cross-resistance, but it does not prove a common resistance mechanism. This pattern is biologically plausible because spinosad and spinetoram belong to the same insecticide class and act through closely related nicotinic acetylcholine receptor-associated pathways [30,31]. Studies in *F. occidentalis* have linked spinosyn resistance to changes involving the nAChR α6 subunit, including α6 knockout or α6-associated sequence variation [32,33]. Nevertheless, the correlation observed in the present study should not be treated as direct molecular proof of the same mechanism in *M. usitatus*. The cross-resistance suggested here is based on bioassay phenotypes. Further work should test whether α6 variation, altered expression of receptor subunits, detoxification enzymes, or other resistance mechanisms contribute to the observed susceptibility patterns. For resistance management, the current evidence is already sufficient to indicate that spinosad and spinetoram should not be used as substitutes for each other in rotation programs.

Compared with acetamiprid and spinetoram, emamectin benzoate and chlorfenapyr showed lower overall resistance levels, but neither should be considered a resistance-free option. Emamectin benzoate reached moderate resistance in the southern populations by 2025, whereas Haikou and Wuzhishan remained at lower resistance levels. Chlorfenapyr also showed clear regional resistance accumulation. All monitored populations reached moderate resistance by 2025, with the highest RR values recorded in Ledong and Sanya. Chlorfenapyr has a mode of action distinct from neonicotinoids and spinosyns, and it has been used as an alternative compound in resistance-management programs for some pest systems [31,34]. However, the present data do not support describing chlorfenapyr as a core or highly reliable rotational insecticide for *M. usitatus* in Hainan. It may still be included as one component of a rotation scheme, but its use should be guided by local susceptibility data. Transcriptomic evidence from *M. usitatus* field populations has suggested that detoxification-related genes may be involved in resistance to several insecticides [24], and this type of mechanism may also influence future changes in susceptibility to emamectin benzoate and chlorfenapyr.

The resistance patterns observed here point to the need for region-specific insecticide resistance management in Hainan cowpea production. In southern areas where acetamiprid resistance has reached high levels, continued routine use of this compound is likely to further reduce its field value. Spinosad and spinetoram still have useful bioassay activity, but their significant susceptibility correlation means that they should not be applied consecutively or rotated with each other as independent alternatives. Chlorfenapyr and emamectin benzoate can be used as rotation components only with restricted frequency and continued monitoring. These recommendations are consistent with national resistance-monitoring guidance and broader IRM principles that emphasize rotation among different modes of action, reduced selection pressure, and regular susceptibility surveillance [28,31,35]. Chemical control should also be combined with pest monitoring, optimized application timing, biological control, and other compatible IPM practices for *M. usitatus* [11,36]. Because this study was based on laboratory bioassays, future work should link LC_50_ shifts with field efficacy, application history, and molecular diagnostic data. This will help determine whether the current phenotypic resistance patterns translate into reduced control performance under field conditions.

## 5. Conclusions

This study revealed clear regional variation in insecticide resistance of *M. usitatus* field populations in Hainan from 2023 to 2025, with southern populations generally showing higher LC_50_ and RR values than those from Haikou and Wuzhishan. Acetamiprid, the tested neonicotinoid compound, showed the greatest reduction in susceptibility, with high-level resistance detected in Ledong, Sanya, and Lingshui by 2025. Spinosyn resistance also increased during the monitoring period, and the significant positive correlation between spinosad and spinetoram responses provided phenotypic evidence of cross-resistance within this insecticide class. However, the relatively low absolute LC_50_ values of spinosad and spinetoram suggest that these compounds may still retain useful bioassay activity when used carefully. Chlorfenapyr and emamectin benzoate also showed regional resistance accumulation and should be used as monitored rotation components rather than unrestricted alternatives. Resistance management should reduce reliance on acetamiprid, avoid consecutive or interchangeable use of spinosad and spinetoram, and strengthen region-specific monitoring within an IPM framework.

## Figures and Tables

**Table 1:** Collection of information on the field populations of *M. usitatus* in Hainan.

Population	Year	Location	Coordinates
Haikou	2023–2025	Xinpo Town	N: 19.7622°, E: 110.3616°
Wuzhishan	2023–2025	Maoyang Town	N: 18.9528°, E: 109.5044°
Ledong	2023–2025	Huangliu Town	N: 18.5087°, E: 108.8336°
Lingshui	2024–2025	Wenluo Town	N: 18.5081°, E: 109.9650°
Sanya	2023–2025	Yacheng Town	N: 18.3736°, E: 109.1517°

**Table 2:** Resistance monitoring of field populations of *M. usitatus* to emamectin benzoate.

Insecticide	Population	Slope ± SE	LC_50_ (95% FL ^a^) (mg·L^−1^)	χ^2^	*df* ^b^	RR ^c^
Emamectin Benzoate	Laboratory strain	1.581 ± 0.195	4.21 (3.36–5.31)	4.577	13	1.00
	Haikou23	1.569 ± 0.181	5.11 (3.86–6.60)	2.594	13	1.21
	Haikou24	1.307 ± 0.127	15.87 (10.79–22.66)	14.234	13	3.77
	Haikou25	1.515 ± 0.179	26.21 (20.46–33.21)	0.839	13	6.23
	Wuzhishan23	1.296 ± 0.144	12.16 (9.35–15.65)	7.144	13	2.89
	Wuzhishan24	1.150 ± 0.117	14.74 (8.76–23.33)	19.878	13	3.50
	Wuzhishan25	1.048 ± 0.169	32.33 (18.18–46.28)	7.002	13	7.68
	Ledong23	1.873 ± 0.193	63.92 (52.46–76.29)	11.145	13	15.18
	Ledong24	2.418 ± 0.213	103.33 (87.78–121.77)	13.4	13	24.54
	Ledong25	1.245 ± 0.168	128.19 (89.57–194.16)	21.665	13	30.45
	Lingshui24	1.080 ± 0.113	55.35 (34.62–95.50)	13.618	11	13.15
	Lingshui25	1.130 ± 0.147	106.54 (71.63–167.72)	11.899	13	25.31
	Sanya23	1.251 ± 0.134	32.70 (12.96–56.48)	18.348	13	7.77
	Sanya24	1.432 ± 0.172	89.14 (70.88–111.13)	11.303	13	21.17
	Sanya25	2.642 ± 0.271	115.56 (99.44–133.76)	6.727	13	27.45

Note: The number of each strain was about 300; ^a^ FL: Fiducial limit; ^b^ *df*: Degrees of freedom; ^c^ RR: Resistance ratio = field strain LC_50_/laboratory strain LC_50_.

**Table 3:** Resistance monitoring of field populations of *M. usitatus* to acetamiprid.

Insecticide	Population	Slope ± SE	LC_50_ (95% FL ^a^) (mg·L^−1^)	χ^2^	*df* ^b^	RR ^c^
Acetamiprid	Laboratory strain	1.860 ± 0.209	6.16 (4.57–8.05)	19.589	13	1.00
	Haikou23	1.913 ± 0.191	66.49 (54.84–79.11)	5.613	13	10.79
	Haikou24	2.010 ± 0.214	125.30 (97.12–161.59)	18.937	13	20.34
	Haikou25	1.992 ± 0.203	283.95 (233.44–337.06)	8.93	13	46.10
	Wuzhishan23	2.109 ± 0.202	196.92 (160.47–239.25)	15.945	13	31.97
	Wuzhishan24	2.716 ± 0.231	243.12 (206.65–286.93)	15.857	13	39.47
	Wuzhishan25	1.375 ± 0.158	255.59 (158.23–361.58)	21.898	13	41.49
	Ledong23	1.451 ± 0.175	353.65 (284.87–452.63)	7.283	13	57.41
	Ledong24	1.806 ± 0.192	535.10 (427.02–646.73)	10.59	13	86.87
	Ledong25	0.981 ± 0.162	1838.63 (1336.91–2869.44)	12.522	13	298.48
	Lingshui24	1.689 ± 0.200	530.55 (427.66–660.90)	10.344	13	86.13
	Lingshui25	2.470 ± 0.248	802.36 (682.28–959.00)	7.600	13	130.25
	Sanya23	1.682 ± 0.182	306.26 (253.32–375.59)	8.941	13	49.72
	Sanya24	2.280 ± 0.237	524.52 (439.55–624.76)	7.018	13	85.15
	Sanya25	2.464 ± 0.245	859.93 (687.55–1124.71)	20.778	13	139.60

Note: The number of each strain was about 300; ^a^ FL: Fiducial limit; ^b^ *df*: Degrees of freedom; ^c^ RR: Resistance ratio = field strain LC_50_/laboratory strain LC_50_.

**Table 4:** Resistance monitoring of field populations of *M. usitatus* to chlorfenapyr.

Insecticide	Population	Slope ± SE	LC_50_ (95% FL ^a^) (mg·L^−1^)	χ^2^	*df* ^b^	RR ^c^
Chlorfenapyr	Laboratory strain	1.912 ± 0.242	11.43 (7.24–18.64)	18.825	13	1.00
	Haikou23	0.806 ± 0.168	208.10 (109.32–441.12)	21.038	16	18.21
	Haikou24	1.380 ± 0.173	301.19 (227.18–379.64)	8.763	13	26.35
	Haikou25	2.014 ± 0.201	321.76 (254.66–395.45)	16.398	13	28.15
	Wuzhishan23	0.913 ± 0.113	86.96 (43.00–164.13)	19.87	13	7.61
	Wuzhishan24	1.802 ± 0.226	193.78 (41.84–244.46)	26.636	16	16.95
	Wuzhishan25	1.667 ± 0.191	285.64 (225.48-–348.72)	11.316	13	24.99
	Ledong23	0.894 ± 0.117	225.73 (145.30–384.39)	22.03	13	19.75
	Ledong24	0.965 ± 0.166	531.68 (356.92–1032.36)	15.713	13	46.52
	Ledong25	2.015 ± 0.213	956.11 (706.90–1211.36)	18.595	13	83.65
	Lingshui24	1.883 ± 0.194	123.90 (101.45–147.97)	10.427	13	10.84
	Lingshui25	1.412 ± 0.178	349.60 (261.86–449.87)	13.519	13	30.59
	Sanya23	1.912 ± 0.242	285.77 (180.99–465.93)	18.825	13	25.00
	Sanya24	1.879 ± 0.180	536.13 (421.87–654.26)	6.867	16	46.91
	Sanya25	1.045 ± 0.089	736.30 (554.19–978.66)	13.786	16	64.42

Note: The number of each strain was about 300; ^a^ FL: Fiducial limit; ^b^ *df*: Degrees of freedom; ^c^ RR: Resistance ratio = field strain LC_50_/laboratory strain LC_50_.

**Table 5:** Resistance monitoring of field populations of *M. usitatus* to spinosad.

Insecticide	Population	Slope ± SE	LC_50_ (95% FL ^a^) (mg·L^−1^)	χ^2^	*df* ^b^	RR ^c^
Spinosad	Laboratory strain	2.123 ± 0.341	0.35 (0.22–0.53)	14.234	10	1.00
	Haikou23	1.147 ± 0.165	1.45 (1.11–2.00)	3.467	13	4.14
	Haikou24	1.179 ± 0.157	1.90 (1.13–3.30)	10.767	10	5.43
	Haikou25	1.374 ± 0.173	2.04 (1.23–3.48)	12.419	10	5.83
	Wuzhishan23	1.693 ± 0.180	0.79 (0.65–0.96)	7.616	13	2.26
	Wuzhishan24	0.799 ± 0.138	1.91 (0.79–4.82)	15.804	10	5.46
	Wuzhishan25	1.277 ± 0.124	2.50 (1.57–3.74)	21.203	13	7.14
	Ledong23	1.892 ± 0.196	2.41 (1.82–3.05)	19.194	13	6.89
	Ledong24	2.211 ± 0.201	4.53 (3.69–5.63)	18.865	13	12.94
	Ledong25	2.175 ± 0.199	11.31 (9.67–13.26)	7.857	13	32.31
	Lingshui24	1.743 ± 0.153	4.23 (3.33–5.31)	10.177	13	12.09
	Lingshui25	1.144 ± 0.145	12.97 (8.63–22.44)	9.764	10	37.06
	Sanya23	1.729 ± 0.197	1.84 (1.32–2.36)	16.802	13	5.26
	Sanya24	2.874 ± 0.242	3.48 (3.00–4.04)	13.874	13	9.94
	Sanya25	2.062 ± 0.385	9.05 (5.56–13.18)	12.914	10	25.86

Note: The number of each strain was about 300; ^a^ FL: Fiducial limit; ^b^ *df*: Degrees of freedom; ^c^ RR: Resistance ratio = field strain LC_50_/laboratory strain LC_50_.

**Table 6:** Resistance monitoring of field populations of *M. usitatus* to spinetoram.

Insecticide	Population	Slope ± SE	LC_50_ (95% FL ^a^) (mg·L^−1^)	χ^2^	*df* ^b^	RR ^c^
Spinetoram	Laboratory strain	1.053 ± 0.138	0.09 (0.05–0.15)	11.788	10	1.00
	Haikou23	1.426 ± 0.174	0.80 (0.61–1.11)	16.057	13	8.89
	Haikou24	1.888 ± 0.174	0.79 (0.62–0.99)	15.865	13	8.78
	Haikou25	2.012 ± 0.192	1.39 (1.14–1.71)	15.111	13	15.44
	Wuzhishan23	2.146 ± 0.200	0.82 (0.66–1.00)	17.141	13	9.11
	Wuzhishan24	1.625 ± 0.179	2.94 (2.37–3.58)	5.677	13	32.67
	Wuzhishan25	1.453 ± 0.179	2.79 (1.92–3.71)	17.850	13	31.00
	Ledong23	1.864 ± 0.191	1.82 (1.45–2.42)	16.966	13	20.22
	Ledong24	2.278 ± 0.205	4.16 (3.38–5.09)	18.759	13	46.22
	Ledong25	1.824 ± 0.510	9.97 (6.49–63.83)	20.786	16	110.78
	Lingshui24	1.487 ± 0.144	2.22 (1.68–3.04)	23.500	16	24.67
	Lingshui25	1.239 ± 0.107	7.86 (5.12–132.85)	15.723	10	87.33
	Sanya23	1.659 ± 0.183	1.33 (0.99–1.71)	17.945	13	14.78
	Sanya24	2.541 ± 0.239	2.20 (1.87–2.54)	11.316	13	24.44
	Sanya25	0.926 ± 0.162	4.08 (2.89–6.84)	12.468	13	45.33

Note: The number of each strain was about 300; ^a^ FL: Fiducial limit; ^b^ *df*: Degrees of freedom; ^c^ RR: Resistance ratio = field strain LC_50_/laboratory strain LC_50_.

## Data Availability

The original contributions presented in this study are included in the article. Further inquiries can be directed to the corresponding authors.

## References

[1] Boukar O., Belko N., Chamarthi S., Togola A., Batieno J., Owusu E., Haruna M., Diallo S., Umar M.L., Olufajo O. (2019). Cowpea (*Vigna unguiculata*): Genetics, Genomics and Breeding. Plant Breed..

[2] Sarr A., Bodian A., Gueye M.C., Gueye B., Kanfany G., Diatta C., Bougma L.A., Diop E.A.M.C., Cissé N., Diouf D. (2022). Ethnobotanical Study of Cowpea (*Vigna unguiculata* (L.) Walp.) in Senegal. J. Ethnobiol. Ethnomed..

[3] Kebede E., Bekeko Z. (2020). Expounding the Production and Importance of Cowpea (*Vigna unguiculata* (L.) Walp.) in Ethiopia. Cogent Food Agric..

[4] Tang L.-D., Yan K.-L., Fu B.-L., Wu J.-H., Liu K., Lu Y.-Y. (2015). The Life Table Parameters of *Megalurothrips usitatus* (Thysanoptera: Thripidae) on Four Leguminous Crops. Fla. Entomol..

[5] Guo L.-H., Wu S.-Y., Gong R.-N., Tang L.-D. (2023). Parthenogenesis Affects Interspecific Competition between *Megalurothrips usitatus* and *Frankliniella intonsa* (Thysanoptera: Thripidae) in Changing Environment: Evidence from Life Table Study. J. Econ. Entomol..

[6] Zhang S., Lin Y., Chen J., Lei Z., Niu Y., Li F., Wu S. (2025). Occurrence Patterns of Major Insect Pests on Cowpea and Evaluation of Cowpea Varieties Resistant to *Megalurothrips usitatus* in Hainan. J. Trop. Biol..

[7] Prasada Rao R.D.V.J., Reddy A.S., Reddy S.V., Thirumala-Devi K., Rao S.C., Manoj Kumar V., Subramaniam K., Yellamanda Reddy T., Nigam S.N., Reddy D.V.R. (2003). The Host Range of Tobacco Streak Virus in India and Transmission by Thrips. Ann. Appl. Biol..

[8] Park C.-G., Kim H.-Y., Lee J.-H. (2010). Parameter Estimation for a Temperature-Dependent Development Model of *Thrips palmi* Karny (Thysanoptera: Thripidae). J. Asia-Pac. Entomol..

[9] Whitfield A.E., Ullman D.E., German T.L. (2005). Tospovirus-Thrips Interactions. Annu. Rev. Phytopathol..

[10] He Y., Geng J., Gao Y., Chen Q., Zhou Y., Zhu Z.-R. (2024). Effects of Weather Parameters on the Population Dynamics of *Megalurothrips usitatus* in Cowpea Fields in Sanya, China. Int. J. Trop. Insect Sci..

[11] Zhang S., Jin H., Liu K., Chen Q., Li F., Wu S. (2025). Sustainable Development of Hainan’s Melon and Vegetable Industry: New Strategies for Pest Control. Trop. Plants.

[12] Peng Z., Li M., Guo C., Zheng H., Wu M., Yin F., Xiao Y., Wang H., Kong X., Zalucki M.P. (2025). Field-Based Evaluation of Insecticide Effectiveness on *Megalurothrips usitatus* in Guangdong, China: Implications for Pest Control Strategies. Insects.

[13] Ivey C., De Marchi B.R., Beuzelin J., Soto-Adames F., Hochmuth R., Turechek W.W., Smith H. (2024). Susceptibility to Insecticides of *Megalurothrips usitatus* (Bagnall) and *Frankliniella insularis* (Franklin) (Thysanoptera: Thripidae) Infesting *Lablab purpureus* in Florida. Crop Prot..

[14] Barathi S., Sabapathi N., Kandasamy S., Lee J. (2024). Present Status of Insecticide Impacts and Eco-Friendly Approaches for Remediation-A Review. Environ. Res..

[15] Li F., Jin H., Yao Z., Xian L., Liu K., Wang L., Zhang K., Shi X., Jiang W., Wu S. (2024). A New Optical Practice as an Effective Alternative to Insecticides for Controlling Highly Resistant Thrips. Trop. Plants.

[16] Zhang K., Yuan J., Wang J., Hua D., Zheng X., Tao M., Zhang Z., Wan Y., Wang S., Zhang Y. (2022). Susceptibility Levels of Field Populations of *Frankliniella occidentalis* (Thysanoptera: Thripidae) to Seven Insecticides in China. Crop Prot..

[17] Bielza P., Quinto V., Contreras J., Torné M., Martín A., Espinosa P.J. (2007). Resistance to Spinosad in the Western Flower Thrips, *Frankliniella occidentalis* (Pergande), in Greenhouses of South-Eastern Spain. Pest Manag. Sci..

[18] Bielza P., Quinto V., Fernández E., Grávalos C., Contreras J. (2007). Genetics of Spinosad Resistance in *Frankliniella occidentalis* (Thysanoptera: Thripidae). J. Econ. Entomol..

[19] Lebedev G., Abo-Moch F., Gafni G., Ben-Yakir D., Ghanim M. (2013). High-Level of Resistance to Spinosad, Emamectin Benzoate and Carbosulfan in Populations of *Thrips tabaci* Collected in Israel. Pest Manag. Sci..

[20] Bao W.X., Narai Y., Nakano A., Kaneda T., Murai T., Sonoda S. (2014). Spinosad Resistance of Melon Thrips, *Thrips palmi*, Is Conferred by G275E Mutation in *α6* Subunit of Nicotinic Acetylcholine Receptor and Cytochrome P450 Detoxification. Pestic. Biochem. Physiol..

[21] Fu B., Li Q., Qiu H., Tang L., Zeng D., Liu K., Gao Y. (2018). Resistance Development, Stability, Cross-Resistance Potential, Biological Fitness and Biochemical Mechanisms of Spinetoram Resistance in *Thrips hawaiiensis* (Thysanoptera: Thripidae). Pest Manag. Sci..

[22] Shi P., Guo S.-K., Gao Y.-F., Cao L.-J., Gong Y.-J., Chen J.-C., Yue L., Li H., Hoffmann A.A., Wei S.-J. (2020). Variable Resistance to Spinetoram in Populations of *Thrips palmi* across a Small Area Unconnected to Genetic Similarity. Evol. Appl..

[23] Fu B., Tao M., Xue H., Jin H., Liu K., Qiu H., Yang S., Yang X., Gui L., Zhang Y. (2022). Spinetoram Resistance Drives Interspecific Competition between *Megalurothrips usitatus* and *Frankliniella intonsa*. Pest Manag. Sci..

[24] Cao H., Yuan J., Wan Y., Tang Y., Zheng X., Wang J., Qian K., Feng J., Chen S., Zhang Y. (2025). Transcriptome Analysis of Insecticide Resistance Mechanisms in Field Populations of the Bean Flower Thrips, *Megalurothrips usitatus* (Bagnall). Ecotoxicol. Environ. Saf..

[25] Yu H., Wu M., Li S., Li J., Zou X., Guo Z., Wu Q., Zhang Y., Kong X., Xie W. (2024). A Maximum Dose Bioassay to Assess Efficacy of Spinetoram against Cowpea Thrip *Megalurothrips usitatus* in China. Insects.

[26] Su T., Liu H. (2025). History and Future Perspectives of Spinosad for Mosquito Control. Pest Manag. Sci..

[27] Rueda A., Shelton A. (2003). Development of a Bioassay System for Monitoring Susceptibility in *Thrips tabaci*. Pest Manag. Sci..

[28] Ren Z.-J., Wang Y.-P., Qin M., Yang X.-X., Li Y.-P., Yuan H.-Z., Zhang S. (2025). National Monitoring, Assessment, and Management Strategies for Agricultural Pest Resistance in 2024. China Plant Prot..

[29] Zhang K., Chen L., Chen J., Huang H., Liu K., Zhang Y., Yang J., Wu S. (2024). Mutation V65I in the β1 Subunit of the Nicotinic Acetylcholine Receptor Confers Neonicotinoid and Sulfoxaflor Resistance in Insects. J. Agric. Food Chem..

[30] Sparks T.C., Wessels F.J., Perry T., Price M.J., Siebert M.W., Mann D.G.J. (2025). Spinosyn Resistance and Cross-Resistance: A 25-Year Review and Analysis. Pestic. Biochem. Physiol..

[31] Sparks T.C., Nauen R. (2015). IRAC: Mode of Action Classification and Insecticide Resistance Management. Pestic. Biochem. Physiol..

[32] Zhang K., Yuan J., Wan Y., Wang J., Zheng X., Zhang Y., Wu S., Liang P., Zhou X., Wu Q. (2023). An Insertion in Intron 3 of *nAChR α6* Subunit Is Associated with Spinosad Resistance in the Western Flower Thrips *Frankliniella occidentalis*. Entomol. Gen..

[33] Mocchetti A., Nikoloudi A.A., Vontas J., De Rouck S., Van Leeuwen T. (2025). CRISPR/Cas9 Knock-out of *nAChR α6* Confers Resistance to Spinosyns in *Frankliniella occidentalis* and Is Associated with a Higher Fitness Cost than Target-Site Mutation G275E. Pestic. Biochem. Physiol..

[34] Raghavendra K., Barik T.K., Sharma P., Bhatt R.M., Srivastava H.C., Sreehari U., Dash A.P. (2011). Chlorfenapyr: A New Insecticide with Novel Mode of Action Can Control Pyrethroid Resistant Malaria Vectors. Malar. J..

[35] Van Leeuwen T., Dermauw W., Mavridis K., Vontas J. (2020). Significance and Interpretation of Molecular Diagnostics for Insecticide Resistance Management of Agricultural Pests. Curr. Opin. Insect Sci..

[36] Tang L.-D., Guo L.-H., Wu J.-H., Zang L.-S. (2023). Thrips in Genus Megalurothrips (Thysanoptera: Thripidae): Biodiversity, Bioecology, and IPM. J. Integr. Pest Manag..

